# Isavuconazole for Treating Invasive Mould Disease in Solid Organ Transplant Recipients

**DOI:** 10.3389/ti.2023.11845

**Published:** 2023-12-15

**Authors:** Jose Tiago Silva, Shahid Husain, José María Aguado

**Affiliations:** ^1^ Unit of Infectious Diseases, University Hospital 12 de Octubre, Instituto de Investigación del Hospital 12 de Octubre (imas12), School of Medicine, Universidad Complutense, Madrid, Spain; ^2^ Centro de Investigación Biomédica en Red de Enfermedades Infecciosas (CIBERINFEC), Instituto de Salud Carlos III (ISCIII), Madrid, Spain; ^3^ Department of Transplant Infectious Diseases, Multi-Organ Transplant Program, University Health Network, Toronto, ON, Canada

**Keywords:** isavuconazole, solid organ transplantation, invasive mould disease, invasive fungal infections, invasive aspergillosis

## Abstract

Solid organ transplant (SOT) recipients have a higher risk of developing invasive mould diseases (IMD). Isavuconazole is a novel broad-spectrum azole active against *Aspergillus* spp. and Mucor, well tolerated, with an excellent bioavailability and predictable pharmacokinetics, that penetrates in most tissues rapidly, and has few serious adverse effects, including hepatic toxicity. Contrary to other broad-spectrum azoles, such as voriconazole and posaconazole, isavuconazole appears to show significant smaller drug-drug interactions with anticalcineurin drugs. We have performed an extensive literature review of the experience with the use of isavuconazole in SOT, which included the SOTIS and the ISASOT studies, and published case reports. More than 140 SOT recipients treated with isavuconazole for IMD were included. Most patients were lung and kidney recipients treated for an *Aspergillus* infection. Isavuconazole was well tolerated (less than 10% of patients required treatment discontinuation). The clinical responses appeared comparable to that found in other high-risk patient populations. Drug-drug interactions with immunosuppressive agents were manageable after the reduction of tacrolimus and the adjustment of mTOR inhibitors at the beginning of treatment. In conclusion, isavuconazole appears to be a reasonable option for the treatment of IMD in SOT. More clinical studies are warranted.

## Introduction

Solid organ transplant (SOT) recipients have a significant high risk of developing invasive mould diseases (IMD) due to the impact of the immunosuppressive drugs on the patient’s immune response [1]. IMD in SOT are mainly caused by *Aspergillus* spp., followed by mucormycosis (zygomycosis), *Fusarium*, *Scedosporium*, and by dematiaceous fungi (dark molds) [2]. Lung transplant recipients have a higher risk for developing invasive aspergillosis (IA) (tracheobronchitis and pulmonary aspergillosis [IPA]) due to specific characteristics related to this transplant: higher rate of pre-transplant colonization, airway ischemia, impaired ciliary function, blunted cough reflex, and denervation injury [3]. Other known risk factors for IMD are post-transplantation renal replacement treatment, cytomegalovirus infection, treatment for acute rejection, mechanical ventilation, extracorporeal membrane oxygenation (ECMO), and liver re-transplantation or transplantation due to fulminant hepatic failure [4, 5]. The morbidity and mortality associated with these infections is extremely high. In most cases, diagnosis is made after invasive procedures, and treatment usually requires a prompt and multidisciplinary treatment, requiring surgical resection of the infection site in some cases [6].

The treatment of choice for IA is voriconazole [7, 8], but the potential hepatotoxicity associated to the drug, as well as its inhibition of cytochrome CYP3A4 and the consequent elevation of serum levels of immunosuppressive drugs (tacrolimus, cyclosporine, and sirolimus/everolimus), makes its use problematic in SOT recipients [9]. Liposomal amphotericin B is the antifungal of choice for the treatment of mucormycosis, while posaconazole is used as a second-line drug [10]. However, the increased risk of nephrotoxicity associated with amphotericin B [11] and the interactions between posaconazole and immunosuppressive drugs [12], entails that the administration of these antifungals in SOT is not without risk.

Isavuconazole (Cresemba^®^; Pfizer, New York City, United States) is the drug most recently incorporated into the azoles. The drug shows predictable pharmacokinetics, good tolerance and few adverse effects (a low incidence of gastrointestinal symptoms, headache, peripheral edema, and dose-dependent shortening of the QT interval have been described), excellent oral bioavailability and good diffusion to tissues, including the central nervous system [13]. Moreover, the intravenous formulation of isavuconazole does not contain the excipient sulfobutyl ether *β*-cyclodextrin sodium (SBECD), which would facilitate its use in patients with moderate or severe renal insufficiency. Experimental animal studies have also confirmed the synergistic action between isavuconazole and micafungin in the treatment of IPA [14].

We have performed an extensive literature review concerning the use of isavuconazole in SOT, and described the most frequent side-effects, clinical response and mortality when isavuconazole was prescribed for the treatment of an IMD.

## Patients and Methods

We conducted a computer-based PubMed (Medline) search with the MeSH (Medical Subject Headings) terms “Isavuconazole,” “Solid Organ Transplantation,” “Infection Fungal Infection” or “Invasive Mould Disease” to identify published literature between March 2015 and June 2023 pertaining the clinical use of isavuconazole in SOT for the treatment of IMD. We searched for articles written in English language.

We have especially focused on the adjustments made on the maintenance immunosuppressive regimen during isavuconazole treatment, the rate of adverse events associated to the antifungal drug, and the clinical response of the IMD to the treatment with isavuconazole.

Case reports, and prospective or retrospective clinical studies which included SOT recipients treated with isavuconazole for an IMD were considered. Articles for which data could not be extracted from the published results were not considered.

We have defined “end of follow-up period” as the last follow-up visit described in the revised articles. “IMD-related mortality” was defined as all demise which resulted of the IFI for which the patient was being treated. For prospective or retrospective clinical studies, IMD-related mortality was determined based on the rates presented by the authors of the articles. For case reports, we have carefully reviewed all the clinical cases, and determine, in case of the patient’s demise, if this was related to the IMD for which the patient was being treated with isavuconazole.

### Statistical Analysis

Quantitative variables are shown as mean (or median) ± standard deviation (or interquartile range [IQR]), whereas qualitative variables are depicted as absolute and relative frequencies. The statistical analysis was carried out using SPSS v. 23.0 (IBM Corp, Armonk, NY).

## Results

### Clinical Characteristics of the Study Population

**FIGURE 1:**
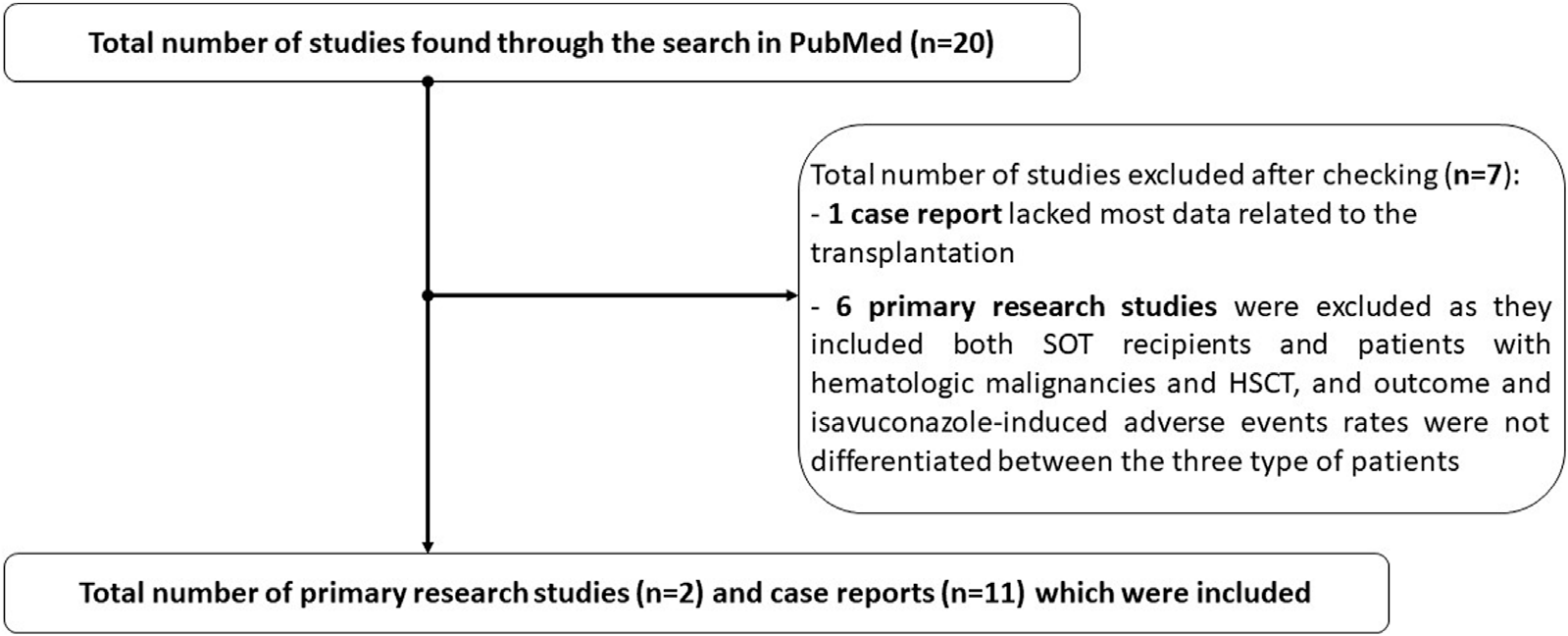
Flowchart of the study.

We identified 20 studies which included at least one SOT recipient who received isavuconazole as treatment for an IMD (Figure 1). Overall, 13 studies met the inclusion criteria, including one prospective observational study which described 53 SOT recipients treated with isavuconazole for fungal infections [15], one multicenter retrospective study with 81 SOT recipients with proven or probable IMD treated with isavuconazole for ≥24 h as first-line or salvage therapy [16], and eleven case reports [17–27]. One case report was not included as it lacked most data related to the transplantation and six studies were excluded as they included both SOT and patients with hematologic malignancies and stem cell transplantation. The key features of the included studies are available in the Supplementary Table S1.

A total of 145 SOT recipients were included (Table 1). Mean age at diagnosis of IMD was 58.3 ± 2.9 years, and 36.6% of recipients were female. Lung transplant accounted for 48.3% of recipients, followed by kidney transplant (24.8%) and liver transplant (13.8%). Median time from the transplantation and diagnosis of IMD was 174 days (IQR 122–174). The majority of recipients were receiving corticosteroids (94.5%), tacrolimus (91.0%) and mycophenolate mofetil/mycophenolate sodium (78.6%) as maintenance immunosuppressive regimen. Interestingly, 13.8% of patients were receiving an mTOR inhibitor. Also noteworthy, 40.0% of patients were receiving anti-mould prophylaxis previous the diagnosis of IFI, especially nebulized amphotericin B (34.5%) (Table 1).

**TABLE 1:** Baseline and clinical characteristics of the 145 SOT recipients included.

	Recipients included (*n* = 145)
Age at diagnosis of IMD, mean ± SD, y	58.3 ± 2.9
Female gender, n (%)	53 (36.6)
Type of transplant, n (%)^a^	
Lung transplant	70 (48.3)
Kidney transplant	36 (24.8)
Liver transplant	20 (13.8)
Combined liver-kidney	1 (0.7)
Sequential pancreas after kidney	1 (0.7)
Heart transplant	14 (9.6)
Small bowel/multivisceral	4 (2.8)
Maintenance immunosuppressive regimen, n (%)	
Corticosteroids	137 (94.5)
Tacrolimus	132 (91.0)
Cyclosporine	4 (2.8)
MMF/MPS	114 (78.6)
Azathioprine	5 (3.4)
Everolimus	13 (9.0)
Sirolimus	7 (4.8)
Posttransplant complications, n (%)	
ECMO	9 (6.2)
COVID-19 infection	13 (9.0)
Use of prophylaxis previous diagnosis, n (%)	58 (40.0)
Echinocandin	8 (5.5)
Nebulized amphotericin B	50 (34.5)

COVID-19, coronavirus disease 2019; ECMO, extracorporeal membrane oxygenation; MMF/MPS, mycophenolate mofetil/mycophenolate sodium.

^a^ One recipient received a combined single sequential lung and liver transplantation.

### Clinical Characteristics of the Invasive Fungal Infections and Efficacy of Isavuconazole Therapy

The most common IFI in our review was produced by *Aspergillus* spp. (82.1%), followed by mucormycosis (9.7%), *Alternaria* (2.1%), *Lomentospora prolificans* (0.7%), *Cladophialophora bantiana* (0.7%), *Diaporthe spp* (0.7%) and *Purpureocillium lilacinus* (0.7%) (Table 2). It’s import to mention that up to four patients (2.8%), who were included in the ISASOT study [15] and that received treatment with isavuconazole, were diagnosed with an IFI which was not produced by a mould. The most common presentation was fungal pneumonia (44.8%) followed by tracheobronchitis (22.1%). Approximately 8.3% of the patients presented disseminated fungal infection.

**TABLE 2:** Clinical characteristics of the fungal invasive infections.

	Recipients included (*n* = 145)
Time from transplantation to IFI, median (IQR), d	174 (122–174)
Moulds isolated, n (%)^a^	
*Aspergillus*	119 (82.1)
Mucormycosis	14 (9.7)
*Alternaria*	3 (2.1)
*Lomentospora prolificans*	1 (0.7)
*Cladophialophora bantiana*	1 (0.7)
*Diaporthe* spp.	1 (0.7)
*Purpureocillium lilacinus*	1 (0.7)
Type of fungal infection^b^	
Tracheobronchitis	32 (22.1)
Fungal pneumonia	65 (44.8)
Bronchial anastomotic infection	2 (1.4)
Mycetoma	6 (4.1)
Cutaneous infection	3 (2.1)
Disseminated fungal infection	12 (8.3)
Osteomyelitis	2 (1.4)
Chronic otitis media	1 (0.7)
Rhino-sinusal-cerebral mould infection	3 (2.1)
Primary gastric	2 (1.4)
Primary colonic mucormycosis	1 (0.7)
Primary hepatic IA	1 (0.7)
Primary mediastinal IA	2 (1.4)
Skin and deep soft tissues infection	5 (3.4)
Isolation in donor	2 (1.4)
Post-traumatic wound	1 (0.7)
No proven or probable FI	6 (4.1)
First line-therapy with isavuconazole, n (%)	98 (67.6)
Previous antifungal treatment, n (%)^c^	47 (32.4)
Reasons to stop previous treatment, n (%)	
IV-to-oral switch and avoiding interactions	14 (9.6)
No previous clinical response	11 (7.6)
Switch according to antifungal susceptibility	4 (2.8)
Adverse events with previous treatment	17 (11.7)
Clinical response at last clinic follow-up visit^d^	80 (55.2)
All-cause mortality at last clinic follow-up visit^d^	52 (35.9)
IFI-related mortality^e^	23 (15.9)

IA, invasive aspergilosis; IFI, infection fungal infection; IQR, interquartile range.

^a^ Four SOT, recipients received isavuconazole for an IFI, which was produced by a yeast, whereas in one case, isavuconazole was prescribed for an unidentified new mould species.

^b^ One patient in the ISASOT study was treated for a fungal tracheobronchitis and a subcutaneous infection at the same time.

^c^ Isavuconazole was added to an ongoing lipid complex amphotericin therapy in 1 recipient.

^d^ In the ISASOT study the last follow-up visit was performed 90 days after the end of treatment, whereas in the SOTIS, study the clinical response was evaluated 12 weeks after the initiation of isavuconazole. In the case reports, follow-up spanned from 45 days to 12 months.

^e^ 2 patients died from a fungal pneumonia, 1 from a disseminated aspergillosis, 1 from a disseminated mucormycosis and 1 from a disseminated *C. bantiana* infection, with central nervous system involvement. Detailed data of the 18 cases of IFI-related mortality included in the SOTIS study was not available.

Isavuconazole was prescribed as first line-therapy in 67.6% of recipients, whereas 32.4% of patients had already started an antifungal treatment. The most common reasons to perform a change to isavuconazole were adverse events associated with the first antifungal drug (11.7%), intravenous-to-oral switch and avoid interactions (9.6%), and absence of a clinical response (7.6%). In a specific patient, isavuconazole was added to liposomal amphotericin B as treatment for a mucormycosis.

At the last clinic follow-up visit, approximately 55.2% of patients presented a clinical response to the isavuconazole treatment (Table 2). All-cause mortality and IMD-related mortality was available in all of the 13 included studies. Overall, the all-cause mortality was of 35.9%, with an IFI-related mortality of 15.9%.

### Safety Outcomes

Approximately 29.7% of patients were diagnosed with an isavuconazole-related adverse event (Table 3). The most common adverse events were liver enzyme elevation (18.6%), myopathy (5.5%) and nausea and vomiting (4.1%). No cases of QT shortening were diagnosed. Noteworthy, only 9.0% of patients required premature discontinuation of isavuconazole due to an adverse event (Table 3).

**TABLE 3:** Isavuconazole-related adverse events.

	Recipients included (*n* = 145)^a^
Total number of patients with TEAE, n (%)	43 (29.7)
Type of TEAE, n (%)	
Liver enzyme elevation	27 (18.6)
Myopathy	8 (5.5)
Nausea and vomiting	6 (4.1)
Neurologic or visual disturbances	4 (2.8)
Fatigue	3 (2.1)
Diarrhea	3 (2.1)
Electrolyte disturbance	1 (0.7)
Weight loss	1 (0.7)
Hyporexia	1 (0.7)
Acute renal failure	1 (0.7)
Sinus tachycardia	1 (0.7)
Tacrolimus overdose	1 (0.7)
TEAE requiring premature discontinuation of isavuconazole, n (%)^a^	13 (9.0)

TEAE, treatment-emergent adverse event.

^a^ Isavuconazole was stopped due to hepatoxicity (4 recipients), gastrointestinal disturbances (3 patients), fatigue (2 cases), myopathy (2 patients), neurological adverse event (1 patient) and due to an isavuconazole-induced diarrhea, which promoted tacrolimus overdose and acute renal failure, followed by multiple episodes of sinus tachycardia (1 recipient).

### Dose Adjustment and TDM of Immunosuppressive Agents

Tacrolimus was adjusted in 99 of the 132 patients who were receiving the immunosuppressive drug (75.0%) (Table 4). mTOR inhibitors were adjusted in 60% of patients who were receiving these immunosuppressors (Table 4). A total of 14 recipients were able to concomitantly receive an mTOR inhibitor and isavuconazole.

**TABLE 4:** Dose adjustments of tacrolimus and mTOR inhibitor agents after initiating isavuconazole.

	Recipients included (*n* = 145)
Tacrolimus, n (%)	
Any dose adjustment, n (%)	99/132 (75.0)
mTOR inhibitor	
Any dose adjustment, n (%)	12/20 (60.0)^a^

^a^ In six patients, the mTOR inhibitor was withdrawn, whereas in six recipients the dose of the immunosuppressive drugs was decreased.

## Discussion

We have performed an extensive literature review which included a total of 145 SOT recipients treated with isavuconazole for an IMD. We observed that isavuconazole appeared to be well-tolerated, and that interactions between isavuconazole and the immunosuppressive drugs were manageable. Clinical responses were also similar to that found in other high-risk patient populations.

Isavuconazole was recently approved for the treatment of IA and mucormycosis based in two pivotal trials. In the SECURE trial, a phase 3, double-blind, global multicentre, comparative-group study, patients with suspected invasive mould disease were randomized to receive isavuconazole or voriconazole [28]. A total of 532 patients were enrolled, with 258 patients in each arm. The authors concluded that isavuconazole was non-inferior to voriconazole for the primary treatment of suspected invasive mould disease, and that was better tolerated when compared with voriconazole, with fewer drug-related adverse events (42% vs. 60%, *p* < 0.001) [28]. In the VITAL trial, 37 patients diagnosed with mucormycosis were treated with isavuconazole for a median of 84 days [29]. Patients were matched with up to three contemporaneous FungiScope patients who had received a primary amphotericin B-based treatment for proven or probable mucormycosis. The authors concluded that isavuconazole was active as primary or secondary treatment (refractory or intolerant to other antifungals), with an overall end-of-treatment complete and partial response similar to those associated with liposomal amphotericin B [29]. Interestingly, isavuconazole showed a significantly fewer hepatobiliary adverse events than voriconazole in the SECURE trial (9% vs. 16%, *p* = 0.016), and in the VITAL study less than 10% of enrolled patients experienced an increase in the liver enzymes [28, 29]. Unfortunately, data was still extremely scarce in SOT, since SOT recipients were not included in the SECURE trial, and only one SOT recipient was included in the VITAL trial.

This review included more than 140 SOT patients who received isavuconazole as treatment for an IMD. We have especially addressed clinical response, adverse events and drug-drug interactions.

Although effectiveness was not the main objective of the reviewed studies, we have calculated a clinical response and an all-cause mortality at last clinic follow-up of 55.2% and 35.9%, respectively, and an IFI-related mortality of 15.9%. Our results are similar to other published studies in which SOT recipients were primarily treated with other antifungal drugs. A recently published Spanish cohort study (Diaspersot study), which included 85 (67.4%) SOT recipients with IA mostly treated with voriconazole reported a clinical improvement of 54.6%, a global mortality of 34.1% and an attributed mortality of 24.6% at the third month of diagnosis [30]. The Swiss Transplantation Cohort Study, which included 70 patients diagnosed with probable and proven IA that were treated with antifungal drugs different than isavuconazole, described a mortality rate of 22.9% at the third month of IA diagnosis [31]. Finally, a multinational study which included 112 KT recipients diagnosed with pulmonary IA, who were also treated with antifungal drugs different than isavuconazole, reported that 39.3% of patients had died by the third month of diagnosis, a mortality rate similar to the found by us [32].

The rate of isavuconazole-related side effects and the rate of isavuconazole-emergent adverse events which required permanent discontinuation of treatment in our review was in line with the SECURE trial (29.7% vs. 42%, and 9.0% vs. 14%, respectively) [28]. Moreover, the Diaspersot study reported that of the 85 recipients treated with voriconazole, 30 (35.3%) presented some degree of toxicity and 13 (15.3%) required a premature discontinuation of the triazole [30]. These results indicate that isavuconazole could be associated with a lower rate of drug-induced toxicity in SOT recipients than voriconazole (29.7% vs 35.3% and 9.0% vs 15.3%, respectively). Finally, patients in the ISASOT study, who required discontinuation of voriconazole due to adverse events were able to continue treatment with isavuconazole [15]. Therefore, the rate of isavuconazole-related adverse events and the rate of permanent discontinuation of the drug seems to be considerably lower in SOT when compared to voriconazole.

In most patients, the daily dose of tacrolimus was lowered at the beginning of therapy and increased after isavuconazole discontinuation. Afterwards, tacrolimus was managed according to the plasmatic levels’ during the treatment. Some patients receiving mTOR inhibitors at the beginning of isavuconazole were also able to maintain the immunosuppressive drug, with an overall good tolerance. Based on our review, drug–drug interactions between isavuconazole and immunosuppressive agents appear to be reasonably manageable in the daily clinical practice. These results are in line with previously published studies which concluded that the degree of interactions between isavuconazole and immunosuppressive agents is smaller than that reported for other triazole antifungal agents [33], and that, because of significant interpatient variability and between each type of SOT, therapeutic drug monitoring (TDM) of the immunosuppressive drugs is recommend in guiding the drug dosing [34].

There are some limitations of this study that have to be taken into account. As we have previously mentioned, both the SOTIS and the ISASOT studies did not include a parallel comparator group which was treated with a different antifungal drug. Moreover, the ISASOT study included a significant high number of lung transplant recipients (83.0%), who were treated with isavuconazole for a fungal tracheobronchitis (25/53 [47.1%]). It would also have been interesting to determine the rate of combined treatment used in these studies; unfortunately, these data were not fully available. The length of the follow-up was also different in both the studies and in the case reports, and the total duration of the isavuconazole treatment was not described in some of the case reports. Unfortunately, TDM of isavuconazole was only available in eight patients (5.5%). Interestingly, one patient, after a month of therapy, presented isavuconazole trough levels below the therapeutic range. It was decided to increase the daily dose of isavuconazole to 200 mg every 12 h. Isavuconazole blood levels arose to therapeutic range afterwards [16]. Another patient with isavuconazole trough levels of 7.2 mg/L, required the withdraw of the antifungal drug due to multiple side effects [19]. Two retrospective studies which included 55 and 26 SOT recipients that received isavuconazole as prophylaxis, and had TDM performed for both isavuconazole and tacrolimus, concluded that the interaction between these drugs was more significant after liver transplantation, that the impact of isavuconazole on tacrolimus levels varied between individuals and that a moderate interpatient variability in isavuconazole pharmacokinetic parameters could be observed [35, 36]. It should be remarked that, nowadays, isavuconazole TDM is especially recommended in patients who are unresponsive to treatment, who have unexpected toxicity or possible drug-drug interactions, or if the infection is produced by a mould with elevated minimum inhibitory concentration (MIC) or is located in sanctuary sites such as the central nervous system (CNS) [8]. The strength of our study lies in the fact that it describes the majority of published cases using isavuconazole in SOT for the treatment of IMD, including its use in patients with non-*Aspergillus* spp. fungal infections, such as *Alternaria*, *Lomentospora* and mucormycosis.

In conclusion, isavuconazole appears to be a well-tolerated drug in SOT recipients, with clinical responses comparable to that found in other high-risk patient populations, and manageable drug–drug interactions, even with calcineurin and mTOR inhibitors. We consider that isavuconazole could be also an acceptable option in non-*Aspergillus* infections in SOT recipients. More future prospective studies are warranted.
