# Supplementary material for: Isavuconazole for Treating Invasive Mould Disease in Solid Organ Transplant Recipients
Source: Transpl Int. 2023 Dec 15;36:11845. doi: 10.3389/ti.2023.11845 (PMC10754982; doi:10.3389/ti.2023.11845)
Supplement: Supplementary file 1 [file Table1.DOCX]

**Supplementary Methods**

**Table S1.** Summary of key features of the studies and case reports that were included.

| **Authors, year, country** | **Objective** | **Methodology** | **Major Findings** |
| --- | --- | --- | --- |
| Fernández-Ruiz *et al*. (1), 2023, Spain | To investigate the efficacy and safety of isavuconazole as first-line or salvage therapy for post-transplant IMD in SOT, in a real-life clinical practice. | Retrospective, multicenter  cohort study, performed in 10 Spanish transplant centers.  Included of 81 SOT recipients treated with isavuconazole for IA (71) or  mucormycosis (n = 10). | Isavuconazole was well  tolerated, with reasonable drug–drug interactions and acceptable clinical response rates. |
| Monforte *et al.* (2), 2022, Spain | To assess the suitability, safety, and effectiveness of isavuconazole in SOT, in the daily clinical practice | Prospective, observational study conducted at one Spanish transplant center.  Included 53 SOT recipients  treated with isavuconazole for more than 48 hours. | Isavuconazole was well tolerated and appeared to be effective for treating IFI in SOT. |
| Assaf et al. (3), 2020, France | To describe the safety and efficacy of an isavuconazole-based treatment for an *A. fumigatus* sternal osteomyelitis in a cardiac transplant recipient | Case report | Successful isavuconazole treatment for bone and joint aspergillosis |
| Happaerts *et al.* (4), 2022, Belgium | To report an azole-induced myositis in a lung and liver transplant recipient | Case report | Isavuconazole can induce a relapse of myositis in SOT recipients treated with azoles |
| Hernández *et al*. (5), 2021, United States | To report the case of a disseminated *C. bantiana* infection in a renal transplant recipient. | Case report | Patient succumbed to the IFI |
| Lango‑Maziarz *et al.* (6), 2022, Poland | To report the case of a heart  transplant recipient who presented with colon mucormycosis. | Case report | Patient succumbed to the IFI |
| Kim, *et al*. (7), 2015, United States | To report the case of a lung transplant recipient with a mediastinal *A. fumigatus* infection | Case report | Successful management with isavuconazole and tacrolimus TDM |
| Gani, *et al.* (8), 2019, United States | To describe a case of a gastric mucormycosis in a renal transplant patient treated with isavuconazole monotherapy | Case report | Successful treatment |
| Silva *et al.* (9), 2018, Spain | To describe the case of IA in a small bowel transplant recipient | Case report | Isavuconazole could be an option in SOT recipients that do not tolerate voriconazole due to adverse events |
| Maisons *et al.* (10), 2022, France | To report the first case of a heart transplant recipient diagnosed with a subcutaneous nodule by *A. infectoria*, and who was treated with isavuconazole | Case report | Several adverse events related to isavuconazole |
| Trujillo *et al.* (11), 2021, Spain | To describe the case of an IA diagnosed in a kidney transplant, after a COVID-19 infection | Case report | Successful treatment |
| Kabulski *et al.* (12), 2018, United States | To describe the use of isavuconazole in a lung trasplant recipient diagnosed with mucomycosis | Case report | Successful treatment, using isavuconazole and tacrolimus TDM |
| Dalla Gasperina *et al.* (13), 2019, Italy | To report the treatment outcome of a subcutaneous IMD in a kidney transplant recipient who was treated with isavuconazole | Case report | Successful treatment |
| IA: Invasive aspergillosis; IFI: invasive fungal disease; IMD: Invasive mould infection; SOT: solid organ transplantation; TDM: therapeutic drug monitoring | | | |

**References**

1. Fernandez-Ruiz M, Bodro M, Gutierrez Martin I, Rodriguez-Alvarez R, Ruiz-Ruigomez M, Sabe N, et al. Isavuconazole for the Treatment of Invasive Mold Disease in Solid Organ Transplant Recipients: A Multicenter Study on Efficacy and Safety in Real-life Clinical Practice. Transplantation. 2023;107(3):762-73.

2. Monforte A, Los-Arcos I, Martin-Gomez MT, Campany-Herrero D, Sacanell J, Berastegui C, et al. Safety and Effectiveness of Isavuconazole Treatment for Fungal Infections in Solid Organ Transplant Recipients (ISASOT Study). Microbiol Spectr. 2022;10(1):e0178421.

3. Assaf A, Faure E, Sermet K, Loridant S, Leroy J, Goeminne C, et al. Successful treatment of Aspergillus fumigatus sternal osteomyelitis with isavuconazole in a heart transplant recipient. Transpl Infect Dis. 2020;22(5):e13313.

4. Happaerts S, Wieers M, Vander Mijnsbrugge W, Godinas L, Van Raemdonck D, Ceulemans LJ, et al. Azole-Induced Myositis after Combined Lung-Liver Transplantation. Case Rep Transplant. 2022;2022:7323755.

5. Hernandez C, Lawal F. Cerebral and pulmonary phaeohyphomycosis due Cladophialophora bantiana in an immunocompromised patient. IDCases. 2021;25:e01240.

6. Lango-Maziarz A, Kolaczkowska M, Siondalski P, Duda M, Dubowik M, Lango R. Colon mucormycosis with renal spread resistant to lipid complex amphotericin and isavuconazole treatment in a heart transplant recipient. Pol Arch Intern Med. 2022;132(1).

7. Kim T, Jancel T, Kumar P, Freeman AF. Drug-drug interaction between isavuconazole and tacrolimus: a case report indicating the need for tacrolimus drug-level monitoring. J Clin Pharm Ther. 2015;40(5):609-11.

8. Gani I, Doroodchi A, Falkenstrom K, Berry H, Lee W, Mulloy L, et al. Gastric Mucormycosis in a Renal Transplant Patient Treated with Isavuconazole Monotherapy. Case Rep Transplant. 2019;2019:9839780.

9. Silva JT, Torre-Cisneros J, Aguado JM. [Invasive aspergillosis in solid organ transplantation]. Rev Iberoam Micol. 2018;35(4):206-9.

10. Maisons V, Desoubeaux G, Coustilleres F, Lemaignen A, Chesnay A, Doman M, et al. Intricate isavuconazole therapy of a subcutaneous nodule caused by Alternaria infectoria in a heart transplant recipient. J Mycol Med. 2022;32(2):101235.

11. Trujillo H, Fernandez-Ruiz M, Gutierrez E, Sevillano A, Caravaca-Fontan F, Morales E, et al. Invasive pulmonary aspergillosis associated with COVID-19 in a kidney transplant recipient. Transpl Infect Dis. 2021;23(2):e13501.

12. Kabulski GM, MacVane SH. Isavuconazole pharmacokinetics in a patient with cystic fibrosis following bilateral orthotopic lung transplantation. Transpl Infect Dis. 2018;20(3):e12878.

13. Dalla Gasperina D, Lombardi D, Rovelli C, Di Rosa Z, Lepera V, Baj A, et al. Successful treatment with isavuconazole of subcutaneous phaeohyphomycosis in a kidney transplant recipient. Transpl Infect Dis. 2019;21(6):e13197.
